# Efficient delivery of antigen to DCs using yeast-derived microparticles

**DOI:** 10.1038/srep10687

**Published:** 2015-05-29

**Authors:** Ying Pan, Xiaopeng Li, Tianyi Kang, Hui Meng, Zhouli Chen, Li Yang, Yang Wu, Yuquan Wei, Maling Gou

**Affiliations:** 1 State Key Laboratory of Biotherapy and Cancer Center, West China Hospital, West China Medical School, Sichuan University, Chengdu 610041, P. R. China

## Abstract

Some pathogens can be naturally recognized and internalized by antigen presentation cells (APCs) *in vivo*, providing a platform for efficient vaccine delivery. However, the biosafety concerns discourage the clinical applications of live pathogens. Here, yeast-derived microparticles were prepared for cancer vaccine delivery. By chemical treatment of bread yeast, capsular yeast shell (YS) microparticles were obtained. Ovalbumin (OVA), as a model antigen, was conjugated to the surface of YS. Results indicated that these YS microparticles with a uniform size of ~3.4 μm can be recognized and internalized by dendritic cells (DCs). The YS-mediated antigen delivery can enhance the cellular uptake of antigen by DCs, promote the maturation of DCs, and trigger DCs to release immune co-stimulatory molecules. Immunization with YS-mediated antigen can induce an effective immune response against tumor cells *in vivo*, with contributions from both humoral and cellular immunity. This work suggests that yeast shell microparticles as efficient vaccine delivery system has promising applications in cancer immunotherapy.

Cancer is a major cause of mortality worldwide^1^. Immunotherapy that attempts to harness the power and specificity of the immune system to treat disease has promise for cancer therapy. The molecular identification of human cancer-specific antigens allows the development of antigen-specific immunotherapy for cancer^2^. Nevertheless, efficient induction of antigen-specific immunity, especially cellular immunity, still presents many challenges. Dendritic cells (DCs) are at the centre of the immune system owing to their ability to control both immune tolerance and immunity. As the most potent antigen-presenting cells (APCs), DCs are an essential target in efforts to generate therapeutic immunity against cancer^3^,^4^,^5^. Taking advantage of micro- and nanoparticles that can selectively carry cargos into specific cells^6^,^7^,^8^, efficient delivery of antigens to DCs *via* a particulate system provides an important strategy to enhance the immune response^9^,^10^. While rationally designed artificial particulate systems have applications in drug delivery, natural microparticles that range from pathogens to mammalian cells also possess features that are useful for drug delivery^11^. To our knowledge, some pathogen particles can be naturally recognized by APCs and act as vaccine adjuvants^12^. However, direct administration of pathogen particles has potential risks^13^. To overcome these challenges, pathogen-mimetic artificial particles provide an alternative platform for efficient delivery of vaccine, without causing whole pathogen associated risks^14^. Bread yeast is a well-known fungus that can enhance host immunity^15^,^16^. Recombinant yeast has the capacity to target DCs to induce a broad-based cellular immune response, with implications for cancer immunotherapy^17^. In this study, yeast shell is isolated and used as a pathogen-mimetic vaccine delivery platform in potential cancer immunotherapy. This work could inspire the development of novel vaccine delivery systems^18^,^19^,^20^,^21^,^22^,^23^,^24^.

## Results

**Figure 1:**
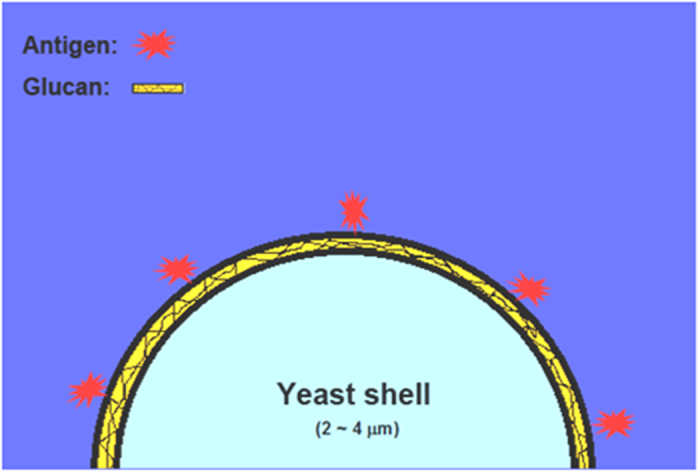
Linking antigen to the surface of yeast shell microparticles for efficient vaccine delivery.

In this work, we developed a bio-inspired vaccine delivery system, which is schematically presented in Fig. 1. Antigen was linked to the surface of capsular yeast shell (YS) microparticles, forming a particulate vaccine which could efficiently generate an antigen-specific immune response against cancer cells *in vivo*.

**Figure 2:**
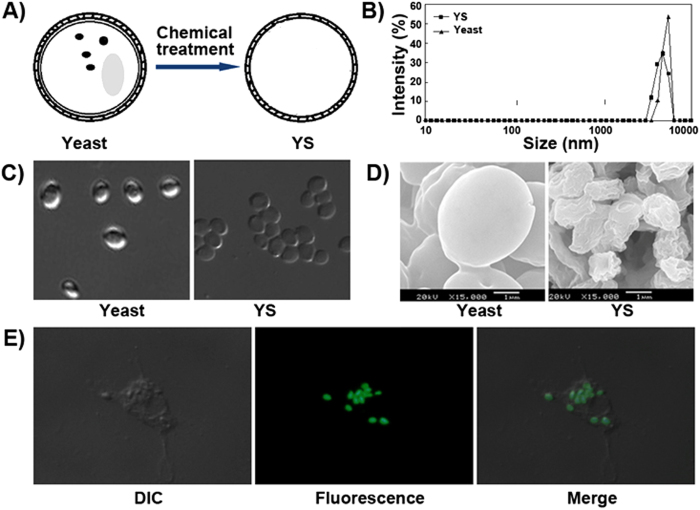
Preparation and characterization of the yeast shell. (**A**) Yeast shell (YS) microparticles were prepared by a chemical treatment of yeast. (**B**) The particle size distribution of yeast and YS. (**C**) The light microscope images of yeast and YS. (**D**) The SEM images of yeast and YS. (**E**) The DC-uptake of FITC-YS.

YS microparticles were prepared by chemical treatment of yeast as shown in Fig. 2A. The YS microparticles had a narrow particle size distribution with a mean particle size of ~3.4 μm (Fig. 2B). Due to the loss of cytoplasmic organelles, the YS microparticles were slightly smaller than whole yeast. As shown in Fig. 2C, light microscopy indicated that YS microparticles had a capsular structure while whole yeast was solid microparticles. The SEM images of YS microparticles and yeast are presented in Fig. 2D. We observed that YS microparticles, unlike whole yeast particles, shrank into particles with a wrinkled surface during the dehydration process because of their hollow structure. Moreover, the cellular internalization of YS was studied *in vitro*. As shown in Fig. 2E, DCs could efficiently uptake YS *in vitro*. But YS, as well as yeast, could not be internalized by cancer cells *in vitro* (data not shown).

**Figure 3:**
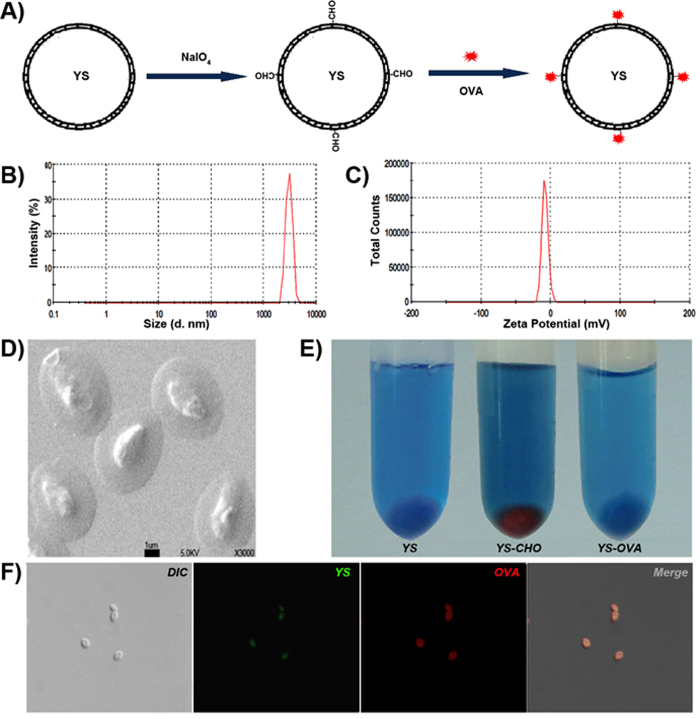
Preparation and characterization of YS-OVA particles. (**A**) Preparation scheme of the YS-OVA. (**B**) The particle size distribution of YS-OVA. (**C**) The zeta potential of YS-OVA. (**D**) The SEM image of YS-OVA. (**E**) Detecting CHO-group on the surface of YS, NaIO_4_ oxidized YS or YS-OVA particles by Filling regents. Red precipitation means the existing of CHO-groups. (**F**) The localization of OVA on YS-OVA particles. YS microparticle was labeled with FITC (green) while OVA was labeled with TRITC (red).

**Figure 4:**
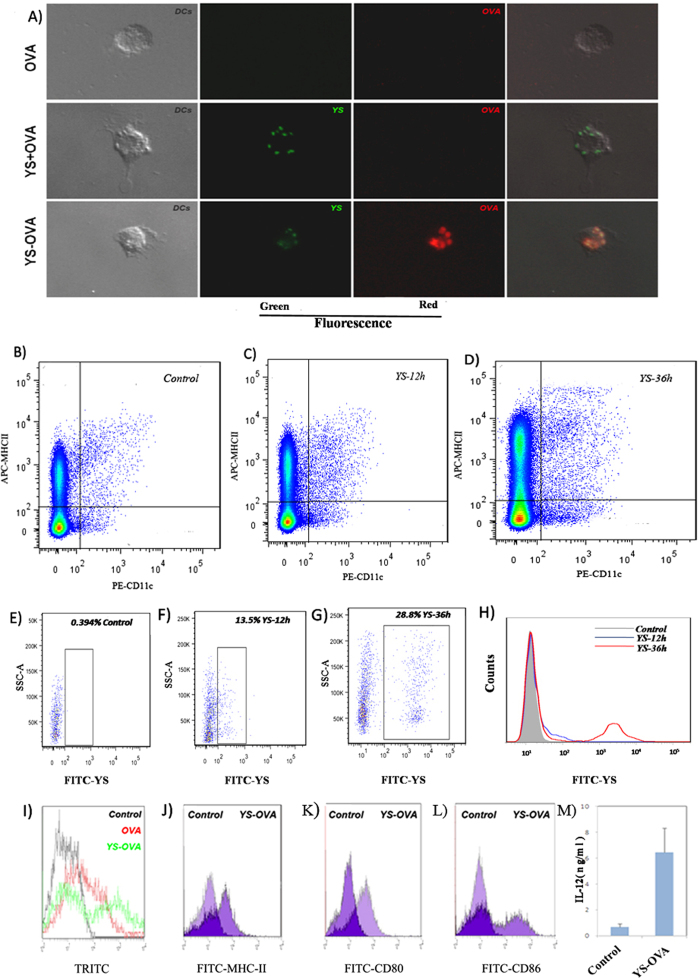
Internalization of YS-OVA by BMDCs. (**A**) 5μg soluble OVA or YS-OVA were incubated with BMDCs for 4 hours. The OVA was coupled to TRITC (red), while YS was coupled to FITC (green). (**B–D**) The dot plots for the *in vivo* uptake of particle by CD11c+ MHCII+ cells in the lymph node at 12 h and 36 h after YS injection or untreated. (**E–G**) Positive ratio of FITC in DCs (zone of square) at 12 h and 36 h after YS injection or untreated was shown. (**H**) An apparent enhancement of FITC fluorescence could be seen along with the time shift (12 h to 36 h). **(I)** Efficiency of BMDC-internalization. (**J–L**) DCs were left unstimulated (zone of left peak shape) or stimulated with YS-OVA (1:2 particle-to-BMDC ratio; zone of right peak shape). Surface expression of MHC-II, CD80 and CD86 was analyzed by FACS. (**M**) Concentration of IL-12 in the supernatant of YS-OVA stimulated BMDCs.

In order to link antigen to YS, we used NaIO_4_ to partially oxidize YS, so that YS possessed an aldehyde group that could react with the primary amine group in protein antigens (schematically presented in Fig. 3A). Ovalbumin (OVA) was used as the model antigen to study the efficiency of YS in delivering antigen. By incubation with NaIO_4_-oxidized YS, OVA was chemically linked to the surface of YS, forming YS-OVA particles with a drug load of 20%. The YS-OVA particles were monodisperse with a mean particle size of ~3.5 μm (shown in Fig. 3B) and had a zeta potential of −8 mV (shown in Fig. 3C). The morphology of YS-OVA determined by SEM is presented in Fig. 3D, in which uniform microparticles can been seen. In testing with Fehling reagent, brick-red precipitate appeared in the oxidized YS group but not in the YS and YS-OVA groups, implying that the aldehyde groups in oxidized YS were exhausted by the reaction with OVA (Fig. 3E). Furthermore, the existence of OVA in the YS-OVA particles was directly observed with fluorescence microscopy. We found that OVA were located on the surface of YS microparticles (Fig. 3F). The capacity of YS to deliver antigens into DCs was evaluated *in vitro*. As shown in Fig. 4A, TRITC-OVA incubated DCs did not exhibit visible TRITC-derived red fluorescence, indicating that DCs could not efficiently internalize pure protein antigen. After incubation with a mixture of OVA and YS, DCs efficiently internalized YS but not OVA, implying that it is difficult to improve DC uptake of antigen via mixing antigen with YS. Nevertheless, DCs internalized both YS and OVA after incubation with YS-OVA efficiently. The ability of DCs to uptake YS particles *in vivo* was examined soon afterwards. 12 and 36 hrs after subcutaneous injection of YS particles, the draining lymph nodes of mouse were taken out respectively. DCs extracted from lymph nodes were marked with MHC-II, CD11c and checked by flow cytometry. The dot plots for the *in vivo* uptake of particle by CD11c+ MHCII+ cells in the lymph node at different time points were shown in Fig. 4B–D.The positive results of green fluorescent validated that YS particles were indeed efficiently taken up by DCs (Fig. 4E–H). This suggests that linking antigen to YS allows efficient delivery of antigen into DCs. Additionally, YS-OVA could not enter cancer cells, avoiding the potential risk of cancer-associated antigen promotion of tumor growth *in vivo* (data not shown). Moreover, the internalization of OVA by DCs was quantitatively studied using flow cytometry. Results indicated that linking OVA to YS enhanced uptake of OVA by DCs (Fig. 4I). Furthermore, cellular uptake of YS promoted the expression of MHC-II, CD80, and CD86 molecules on DCs (Fig. 4J–L). This implies that YS-mediated antigen delivery promotes the maturation of DCs, which significantly enhance the immune response to antigens. In addition, the internalization of YS-OVA triggered DCs to secrete IL-12, which also enhances immunity, especially type I immunity (Fig. 4M).

**Figure 5:**
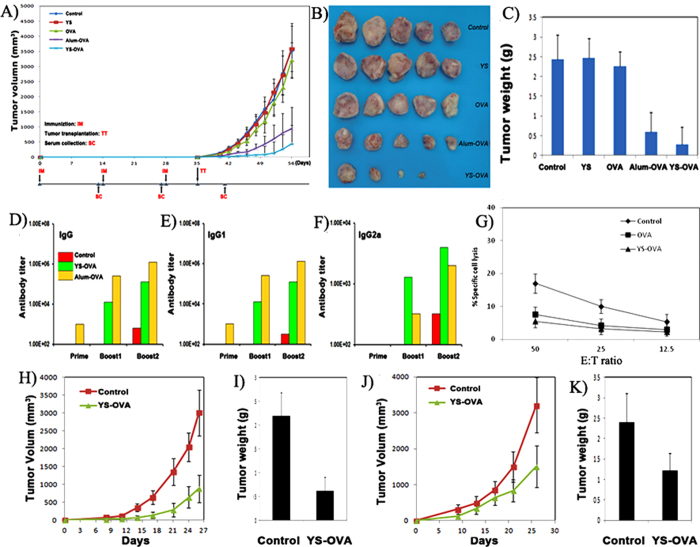
Induction of the protective antitumor immunity. Mice we immunized subcutaneously three times, 7 days after the last time, mice were challenged with 5*10^6^ E.G-7 lymphoma cells. **A**), **B**) and **C**) were the tumor growth curve, image of tumors and weight of tumors in each treatment group (*n* = 5), respectively. **D–F**) Anti-OVA IgG, IgG1 and IgG2a titers as determined by ELISA following each immunization. **G**) *In vitro* cytotoxicity assay. **H**) and **I**) were tumor growth curve and tumor weight of mice after serum adoptive transfer, respectively (*n* = 5). **J**) and **K**) were tumor growth curve and tumor weight of mice after lymphocyte adoptive transfer, respectively (*n* = 5).

The potential application of YS in a cancer vaccine was evaluated *in vivo*. As schematically presented in Fig. 5A, five groups of mice were subcutaneously immunized with 3 doses of normal saline (control), YS, OVA, alum/OVA, or YS-OVA (5 μg OVA per dose), respectively. The mice were then challenged with subcutaneous injection of E.G7-OVA tumor cells. The tumor growth curves of each immunization group indicate that the YS-OVA and alum/OVA vaccines could significantly delay tumor growth (*p < 0.05*), while the tumor growth in the YS and pure OVA vaccine groups was as rapid as that in the control group (*p* > *0.05*). Furthermore, the YS-OVA vaccine was more efficient than alum/OVA in delaying tumor growth (*p < 0.05*). Representative images of tumors in each treatment group are presented in Fig. 5B. The tumors in the YS-OVA vaccine group were significantly smaller than those in the other treatment groups. Moreover, the tumors in each group were weighted, and the results presented in Fig. 5C. The T_w_/C_w_, the ratio of the mean tumor weight in vaccinated mice (T_w_) divided by that of the control group (C_w_), was 0.11 (*p < 0.05*) in the YS-OVA group, 0.25 (*p < 0.05*) in the alum/OVA group, 0.93 (*p* > *0.05*) in the OVA group, and 1.01 (*p* > *0.05*) in the YS group. Compared to alum/OVA, YS-OVA caused a significant reduction in tumor weight (*p < 0.05*). These results indicate that the YS-OVA vaccine could induce an effective protective immune response to E.G-7 tumors *in vivo*.

The mechanism of the YS-OVA vaccine in inducing protective immunity against E.G-7 cancer was studied. OVA-specific serum IgG, IgG1, and IgG2a levels after vaccination with YS-OVA or alum/OVA were determined using ELISA. Similar to alum, YS delivery with antigen induced a high total IgG titer, and booster dosing significantly improved the total IgG titer (Fig. 5D). Among the IgG subtypes, IgG1 levels indicate a Th2 type immune response, while IgG2a titers indicate a Th1 type immune response. As shown in Fig. 5E, IgG1 titers appeared to correlate with total IgG titers, implying that YS as well as alum could trigger the humoral immune response (Fig. 5E). As shown in Fig. 5F, YS-OVA induced higher IgG2a levels than OVA solution and alum/OVA, thus causing a decrease in the IgG1/IgG2a ratio. Furthermore, we studied the roles of OVA-specific antibody and cytotoxic lymphocytes (CTLs) in the anticancer immunity induced by the YS-OVA vaccine. YS-OVA was found having stronger cytotoxic effect on target cells than free OVA (Fig. 5G). As shown in Fig. 5H and Fig. 5I, the adoptive transfer of serum from YS-OVA immunized mice into tumor-challenged mice significantly inhibited tumor growth (*p < 0.05*), implying that YS-OVA generated humoral immunity contributed to its anticancer immunity. Moreover, as presented in Fig. 5J and Fig. 5K, adoptive transfer of T lymphocytes isolated from YS-OVA immunized mice also had some antitumor activity against E.G-7 cancer (*p < 0.05*), suggesting that YS-OVA generated cellular immunity also played a role in its anticancer immunity. Together, these results suggest that both OVA-specific humoral and cellular immunity induced by YS-OVA contributed to the protective immune response against E.G-7 cancer.

## Discussion

Bio-mimetic is an interesting design cue for novel drug delivery systems. Pathogens are naturally microparticles with uniform in particle size, surface morphology and composition. Some pathogen particles can be naturally recognized by APCs and act as vaccine adjuvants. Ali *et al.* designed a pathogen-mimetic polymer that can first release a cytokine to recruit host DCs, and subsequently present cancer antigens and danger signals to activate the resident DCs, with the results of inducing specific and protective antitumor immunity^22^. It is known that the immune system always can accurately respond to particles *via* recognizing their surface^23^,^24^. In this study, pathogen ghost is employed in order to mimic pathogen and target antigens to APCs, with the goal of inducing effective anticancer immunity. Inspired by the yeast-induced immune response, we prepared yeast shell microparticles and demonstrated that linking antigen to the yeast shell can enhance antigen presentation and induce antigen-specific anticancer immunity *in vivo*. Thus, extracting pathogen shell microparticles to deliver antigen may be a useful therapeutic strategy for pathogen-mimetic vaccine delivery. This idea can also be supported by the existing application of bacterial ghosts in vaccine delivery^25^.

Yeast have a cell wall composed mainly of β-glucan with a thickness of ~115 nm^26^,^27^, which is thicker than the cell walls of other pathogens, *e.g.* bacteria. This allows us to readily obtain β-glucan-composed YS microcapsules from yeast using a special chemical method^28^. Nevertheless, loading antigen to YS still has some challenges, because YS is a porous and hydrophilic capsule. In addition to encapsulating antigen in microcapsules, binding antigen on the surface of particles is a reliable strategy to enhance the immune response^29^. Huang *et al.* have attempted to physically encapsulate antigen in β-glucan particles^30^,^31^. In this work, we linked antigen to the surface of YS, which can efficiently induce anticancer immunity *in vivo*. The process is technically feasible and achieves high drug loading. Therefore, linking antigen to the surface of YS is a novel strategy to enhance immunity.

DCs play a key role in both immunity induction and tolerance maintenance. DCs can selectively uptake β-glucan via dectin-1 mediated phagocytosis, followed by activation^32^,^33^,^34^. Moreover, they can internalize the particle-associated proteins and present these antigens to CD8+ T cells (a process known as cross-presentation) more efficiently than if the same extracellular proteins were internalized in a soluble form. In this work, YS was used to mimic the surface properties and particulate nature of yeast. Our results indicated that YS can efficiently enhance antigen presentation by targeting antigens to DCs, promoting the maturation of DCs and triggering DCs to secrete IL-12. Moreover, YS-mediated antigen, but not pure protein antigen, could induce protective immunity against cancer *in vivo*. According to the hallmarks of cancer theory, cellular immunity always has an important role in cancer immunotherapy^35^,^36^. Alum is the clinically used adjuvant for protein antigen vaccines, but it is always insufficient in inducing cellular immunity^37^. Our results indicate that both T lymphocytes and serum isolated from YS-OVA immunized mice had the capacity to inhibit E.G-7 cancer *in vivo*. We suggest that YS-mediated antigen delivery can not only induce humoral immunity but also generate cellular immunity, which contribute to significant YS-mediated antigen-induced anticancer effects. Thus, YS-mediated antigen delivery has promising applications in cancer immunotherapy.

## Conclusion

The capsular yeast shell microparticle is a potential vaccine delivery platform. Linking antigens to the surface of YS microcapsules can enhance the antigen-specific immune response by presenting antigen to DCs. YS microparticles mediated vaccine delivery has promising application in cancer immunotherapy.

## Materials and methods

### Cell Line and Mice

E.G7-OVA lymphoma cell was kept in State Key Lab of Biotherapy (China), and cultured in RPMI-1640 medium with 10% fetal bovine serum, 100 U/mL penicillin, 100 μg/mL streptomycin, 400 μg/mL G418 and cultivated in incubator at 37 °C with 5% CO_2_ atmosphere. Six to eight week old female C57BL/6 mice were purchased from Beijing Huafukang Bio-technology Co. Ltd. (Beijing, China) and raised under pathogen-free conditions. Mice were conformed to the environment for at least one week before experiment. The methods were carried out in accordance with the approved guidelines. All animal experiments were approved by the Institutional Animal Care and Use Committee of West China Hospital of Sichuan University.

### Preparation and Characterization of YS-OVA

Capsular yeast shell was prepared by chemical treatment of yeast^28^. Briefly, 200 g *Sacharomyces cerevisiae* was dispersed in 1 L of 1 M NaOH for 1 h at 80 °C under stirring, followed by wash with distilled water for 3 times. Then, the particles were dispersed in 1 L of HCl solution (pH = 3~4), and incubated at 55 °C for 1 h, followed by wash with distilled water for 3 times. At last, these particles were extracted by 200 mL of isopropanol for 4 times and 200 mL of acetone for 2 times. To link protein antigen to the YS, YS was oxidized by NaIO_4_, followed by incubation with antigen^38^. Briefly, aldehyde YS was prepared by incubation of 50 mg YS with 1.5 mL of NaIO_4_ solution for 30 minutes at room temperature. Then, OVA protein solution and aldehyde YS were mixed at the mass ratio of 2:1, followed by overnight reaction. At last, the YS-OVA particles were washed with PBS for 3 times.

The aldehyde in particles was detected by Fehling reagent which can create brick red precipitation upon reaction with aldehyde group. YS, NaIO_4_ oxidized YS or YS-OVA was incubated with Fehling reagent (solution A and solution B mixed in equal volume) for 5 minutes at 37 °C, followed by centrifugation for observation.

Drug loading (DL) of YS-OVA was characterized by a subtraction method and calculated according to equation (1):


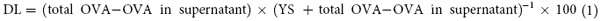


Size distribution and zeta potential were determined by Zetasizer Nano ZS (Malvern Instruments, Worcestershire, UK). Three times of the size and zeta potential of microcapsules and YS-OVA were tested at 25 °C after equilibration for 2 mins.

The morphology of particles was observed under light microscope. A transmission electron microscope (TEM) (H-6009IV, Hitachi, Japan) was utilized to measure the morphology of particles.

### Cellular culture of DCs

Bone marrow cells were obtained and processed by red blood cell lysis buffer for 2 minutes. After washing with RPMI-1640 medium twice, the cells were resuspended in RPMI-1640 medium mixed with GM-CSF and IL-4, final concentration of which is 10 ng/mL respectively. After 48 hours cultivation in incubator at 37 °C with 5% CO_2_ atmospher, all the cells were centrifuged and resuspended with fresh medium. Then, the medium was changed every two days as before. All the suspension cells were collected on day 7 and rich bone marrow-derived dendritic cells were obtained.

### Cellular internalization by DCs

By linking FITC labeled YS to the surface of TRITC labeled OVA, YS(FITC)-OVA(TRITC) was obtained. Day 7 bone marrow derived DCs were incubated with YS(FITC)-OVA(TRITC) containing 5 μg OVA, TRITC labeled soluble OVA (5 μg), and mixture equal yeast-FITC and OVA-FITC for 4 hrs in 6-wells plate. Then cellular uptake of OVA *in vitro* was qualitatively measured under a confocal laser scanning microscope and quantitatively determined by flow cytometry. In addition, YS (50 mg/mL) was administrated subcutaneously to mouse. At two time points (12 h and 36 h), the draining lymph nodes at injection site were taken out and grinded to obtain cell suspension separately. Then the DCs were dyed with MHC-II-APC and CD11c-PE. Uptake of YS-FITC by DCs was tested by flow cytometry.

### DCs maturation and cytokine analysis

To analysis the effects of YS on the DCs mutation, day 7 bone marrow derived DCs (10^5^/mL) were incubated with YS-OVA containing 2 ug OVA or 2 ug soluble OVA. Then cells were subsequently stained with CD11c-PE (BD Pharmingen), MCH-II-FITC (BD Pharmingen), CD80-FITC (BD Pharmingen) and CD86-FITC (BD Pharmingen). These surface markers levels was measured by flow cytometry (BD Immunocytometry Systems) and analyzed by FlowJo software Version 6 (TreeStar). DCs culture supernatants were collected 24 h after culture with samples. The IL-12 cytokine production in the media was analyzed using enzyme-linked immunosorbant assay (ELISA) kits (Becton Dickinson, NJ, USA) according to manufacturer’s directions.

### Tumor challenge

Before tumor challenge, C57 mice (6~8 weeks old, female) were subcutaneously vaccinated with normal saline (negative control), YS, soluble OVA, YS-OVA or Alum-OVA every other week for 3 doses (*n* = 5). One week following the last immunization, each mouse was challenged subcutaneously with 5 × 10^6^ E.G7-OVA lymphoma cells. Tumor size was measured every other day until the tumor volume exceeded 2500 mm^3^ (tumor volume = length × width^2^ × 0.52).

### Detection of Antibody Titers in Serum against OVA

Female C57 mice (6~8 week old) were subcutaneously vaccinated three times contain 5 ug protein include soluble OVA, YS-OVA or mixture of YS and OVA group every other week respectively. The last time was just a week after the second immunization. Two weeks after immunization, blood samples were collected. The titers of OVA specific antibodies were determined by OVA protein ELISA according to manufacturer’s directions.

### *In vitro* cytotoxicity assay

^51^Cr release assay was performed to evaluate the CTL response. Splenocytes obtained from the mice immunized with OVA, YS-OVA or normal saline separately were treated with (Ammonium-Chloride-Potassium) lysing buffer. Each well of microtiter plates was added with a total of 100 ul effector cells and ^51^Cr-labeled target cells at different E:T ratios. After incubation for 4 hours at 37 °C, the supernatant was acquired to detect the radioactivity per minute (RPM). Group of spontaneous release and maximum release were set in parallel. The activity was calculated according to equation (2):


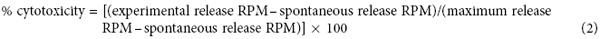


### Adoptive immunotherapy

#### Serum transfer *in vivo*

Mice were immunized every other week for three times (*n* = 5). On day 7 after the third immunization, anti-OVA serum were obtained from the sacrificed mice and mixed together. To access the efficacy of antibodies in antitumor *in vivo*, 100 uL serum was adoptively *i.v* transferred 1day after mice were challenged with 5 × 10^6^ E.G7-OVA cells and then treated every day for 10 days (IgG titer 1:10^5^).

#### Lymphocyte adoptive transfer *in vivo*

Mice were immunized twice a week for three times (*n* = 5). On day 7 after the third immunization, lymphocytes were obtained from the spleen of executed mice. To evaluate the efficacy of lymphocytes in antitumor *in vivo*, 1 × 10^7^ lymphocytes were adoptively *i.v.* transferred 1 day after mice were challenged with 5 × 10^6^ E.G7-OVA cells and then treated every day for 3 days.

### Statistical analysis

Data were expressed as the mean value ± sd. Statistical analysis was performed with one-way analysis of variance (ANOVA) using SPSS software. P values less than 0.05 were considered to be statistically significant.

## Additional Information

**How to cite this article**: Pan, Y. *et al.* Efficient delivery of antigen to DCs using yeast-derived microparticles. *Sci. Rep.* **5**, 10687; doi: 10.1038/srep10687 (2015).
